# Why cost-effectiveness thresholds for global health donors should differ from thresholds for Ministries of Health (and why it matters)

**DOI:** 10.12688/f1000research.131230.2

**Published:** 2024-01-18

**Authors:** Tom Drake, Y-Ling Chi, Alec Morton, Catherine Pitt

**Affiliations:** 1 Department of Global Health, Centre for Global Development, London, UK; 2 Strathclyde Business School, University of Strathclyde, Strathclyde, UK; 3 Department of Global Health and Development, London School of Hygiene and Tropical Medicine, London, UK

**Keywords:** Global health, health financing, aid, priority-setting, cost-effectiveness, threshold

## Abstract

Healthcare cost-effectiveness analysis is increasingly used to inform priority-setting in low- and middle-income countries and by global health donors. As part of such analyses, cost-effectiveness thresholds are commonly used to determine what is, or is not, cost-effective. Recent years have seen a shift in best practice from a rule-of-thumb 1x or 3x per capita GDP threshold towards using thresholds that, in theory, reflect the opportunity cost of new investments within a given country. In this paper, we observe that international donors face both different resource constraints and opportunity costs compared to national decision-makers. Hence, their perspective on cost-effectiveness thresholds must be different. We discuss the potential implications of distinguishing between national and donor thresholds and outline broad options for how to approach setting a donor-perspective threshold. Further work is needed to clarify healthcare cost-effectiveness threshold theory in the context of international aid and to develop practical policy frameworks for implementation.

## Introduction

To maximise population health for the resources available and accelerate progress towards universal health coverage, health systems must make use of evidence to identify which interventions and services to prioritise for investment. The last two decades have seen increasing use of evidence-informed priority-setting to guide resource allocation decisions in many low- and middle-income countries (LMICs). In 2014, the World Health Assembly Resolution WHA67.23 urged countries to consider the use of Health Technology Assessment (HTA) to inform a range of resource allocation decisions from coverage of medicines in formularies to inclusions in benefits packages. ^1^ The use of formal priority-setting processes and methods has intensified in the past decade. ^2^ A *cost-effectiveness threshold* is a decision-rule that can be used alongside a cost-effectiveness analysis (CEA) to determine whether an intervention’s incremental cost-effectiveness ratio (ICER) – that is, the ratio of the additional costs and benefits of an intervention, compared to the next-best alternative – indicates that it would constitute an efficient (i.e., cost-effective) use of scarce resources in a given context. Cost-effectiveness thresholds have gained greater prominence in academic and policy circles in recent years; however, the use of such thresholds is still nascent in many LMICs, in some cases due to the lack of formal processes and institutions to guide coverage decisions. ^3^

This push for the use of CEA and cost-effectiveness thresholds at the national level stands in contrast to methods applied by donors to inform the allocation of Development Assistance for Health (DAH). In recent years, annual DAH has stabilised at around $40bn ($54bn in 2020 including Covid) and represents about a quarter of health spending in low-income countries. ^4^^,^^5^ While some DAH supports research, advocacy, or other catalytic activities, the vast majority supports service delivery, including both financing for specific services (which we focus on in this article) and health system strengthening such as infrastructure investments. ^4^ While many donors have adopted Value for Money (VfM) frameworks ^6^^,^^7^^,^^8^ or other forms of assessments, such as cash benchmarking, ^9^ to our knowledge, few rely substantially on CEA to prioritise their funding allocation to programmes or between countries. The use of explicit cost-effectiveness thresholds by donors is even rarer; with the exception of foundations from the effective altruism movement (i.e., Givewell ^10^ and Open Philanthropy ^11^), we have found none. When such methods are applied, they typically do not consider investments from national decision-makers, which can create issues of fragmentation, lack of alignment with national priorities, displacement of national funds, and duplication of investments. ^12^

There is much debate over what cost-effectiveness thresholds are meant to represent. ^13^^–^^15^ For country thresholds, an emerging consensus is that the threshold should: i) reflect national resource availability, and ii) in application, be equal to the opportunity cost of alternative marginal healthcare spending. ^14^^–^^16^ Two papers from Woods *et al.,* ^17^ and Ochalek *et al*., ^18^ provide initial estimates of national thresholds for 182 countries based on this “supply-side” perspective. Some health economists have suggested that donors should align with national cost-effectiveness thresholds. ^19^

In this piece, we outline the case for distinguishing between donor and national cost-effectiveness thresholds, both in terms of the theoretical basis for the threshold and the potential benefits of clearer separation. We seek to build on and complement existing conversations on the use of cost-effectiveness thresholds in LMICs. ^16^^–^^23^ Our aim is to encourage donors towards actions that promote health maximisation across LMICs, while recognising the importance of other public health goals such as equity and aid effectiveness principles such as country ownership and donor alignment. ^24^

### Why cost-effectiveness thresholds for global health donors should differ from thresholds for Ministries of Health

Donors may distribute DAH to pursue various objectives, which may include reducing preventable mortality and morbidity, protecting their own country’s health (e.g., by reducing global burden of infectious disease) or fostering wider political and economic national interests. ^25^^–^^27^ Where health maximisation is at least one of the purposes of a donor’s DAH, and some form of cost-effectiveness thresholds is therefore potentially relevant, then we argue that the adoption of a separate threshold to national decision-makers becomes useful for the following reasons:

i) **Different resource constraints.** National decision-makers face budget constraints which reflect their country’s overall resources; the extent to which their government is able to mobilise those resources; and the prioritisation of health within national budgets. Donors, by contrast, face wholly separate resource constraints. Country donors’ budgets are constrained by their country’s resources and prioritisation of (health) aid to other countries, while private and multilateral donors’ budgets are constrained by their ability to raise revenue from government and private contributors globally. If cost-effectiveness thresholds reflect the scarcity of resources, then the differing resource constraints imply that differing thresholds should be used.
ii) **Different opportunity costs.** Unlike national institutions, which must prioritise their investments within a single country, global health donors may choose between support for health services across many countries. Therefore, a donor’s opportunity cost of investing in intervention A in country X is not only intervention B in country X, but also intervention C in country Y. For example, while investing in Covid vaccines for the over 60’s in Kenya may represent good value compared with alternative investments in Kenya, it may be more cost-effective to support the roll-out of bed nets in Malawi.

### Why does this matter?

The lack of clarity around differences in donor and national cost-effectiveness thresholds is indicative of the lack of clarity in the decision perspectives and the roles that different actors have in funding healthcare in LMICs. Despite global health financing being a multi-billion-dollar sector where rhetoric on evidence-informed priority-setting is commonplace, many donors lack a clear framework for prioritisation. Collaboration between donors and national institutions in countries which receive DAH is often complex, political, and constantly negotiated for both donors and countries. The result is a fragmented system of financial support that impedes national health leaders in their work to develop an efficient and effective health system. ^28^

At the country level, DAH can alleviate local resource constraints and increase the fiscal space for health and thereby support the provision of health services that otherwise would not be possible. While this often takes the form of “plugging the gaps”, as donors see them, an efficiently allocated package of health services run by countries would imply a certain (hypothetical) cost-effectiveness threshold and additional donor funding would imply an (equally hypothetical) threshold that is higher than the country threshold.

**Figure 1.**
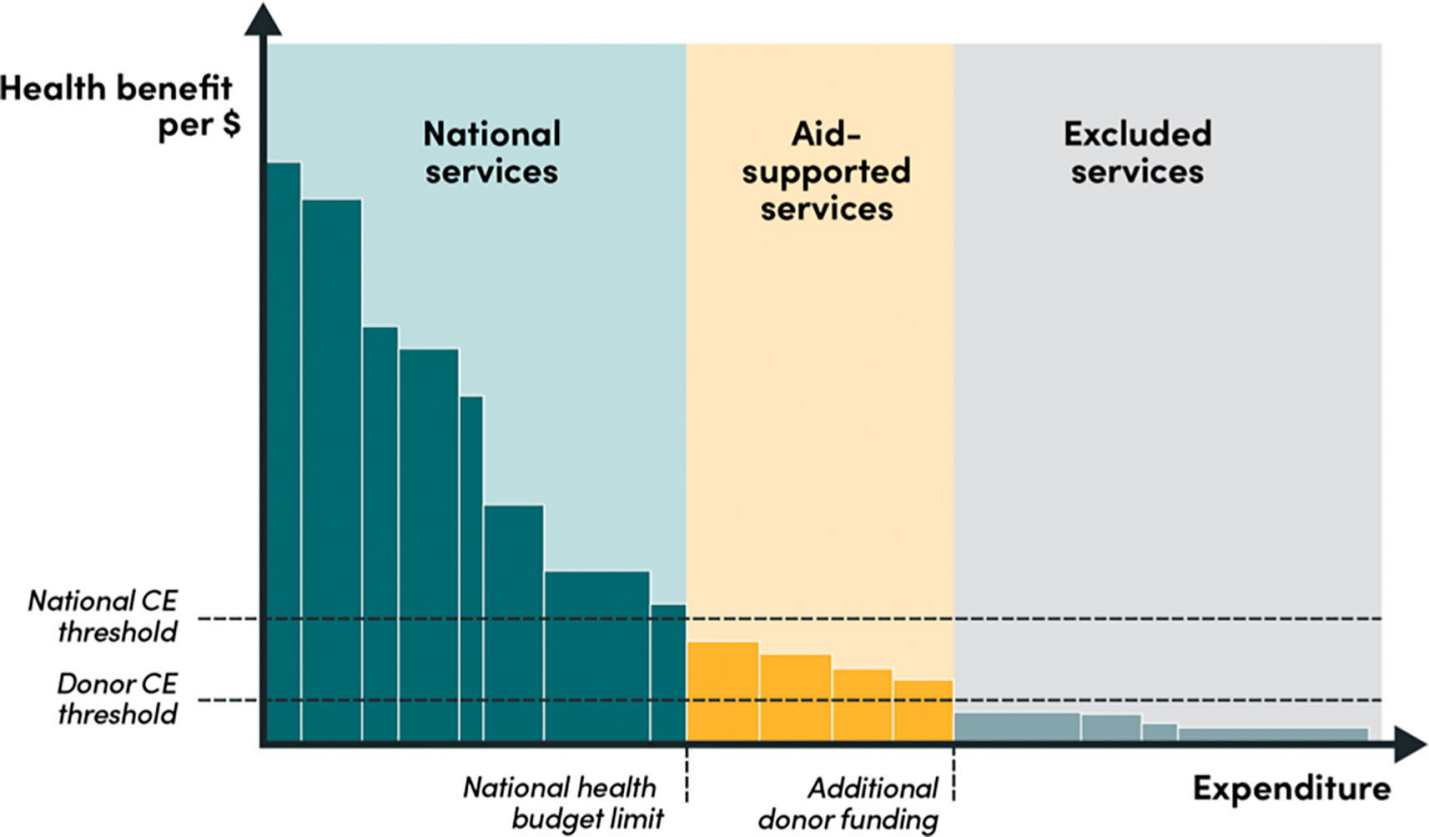
National and donor cost-effectiveness thresholds using the bookshelf metaphor.

The application of separate cost-effectiveness thresholds that reflect the perspective of decision-makers and donors can help to clarify the roles and responsibilities of national vs international funders of health services in LMICs; in other words, it would create a structure for who-should-fund-what. National institutions could design and fund a cohesive core package of the most-cost-effective services up to their national thresholds and “invite” donors to support a top-up package of the next-most-cost-effective services (see Figure 1). Each service – or book in the bookshelf metaphor – could be defined either at the level of the national population or for specific subpopulations for whom the cost-effectiveness differs because of high need, geographical inaccessibility, or other factors. The role of DAH would therefore be *auxiliary*: donors would fund interventions *above* the national threshold, up to their own threshold (we will discuss what this would look like below). In other words, the national cost-effectiveness threshold would represent a *ceiling* for a national payers and a *floor* for donors, below which they would not seek to fund activities in that country.

This approach could address some of the greatest challenges in global health financing. ^12^ First, it could focus national resources towards funding a core package of the most essential services, which could ensure that funding for the provision of key services is not affected by aid volatility. Second, fragmentation of health financing ^29^^,^^30^ (and resulting duplication) could be reduced by a clearer separation of funding responsibilities. Further, the application of separate cost-effectiveness thresholds can avoid displacement of domestic resources by aid. Greater prioritisation of health interventions from a national perspective could be achieved, especially in designing a core package of most essential services – which can maximise the impact of overall health funding, rather than of funding streams operating in silos.

The approach would also empower national institutions to set their own priorities, rather than needing to work within the complex and fragmented financing space created by ad hoc donor support. At present, a significant share of health prioritisation is *de facto* done in donor headquarters and does not necessarily reflect national priorities, particularly in countries where DAH constitutes a large share of total health expenditure. In this framework, as the domestic health budget and cost-effectiveness thresholds increase, health aid is naturally crowded out. Transition from aid or the ending of specific aid programmes does not disrupt the provision of the most cost-effective services. Such transitions to domestic financing will nonetheless continue to require careful design and implementation – possibly including technical assistance and infrastructure investment – to ensure that they are smooth and do not disrupt the delivery of the next-most-cost-effective services. Further work is needed to consider the public financial management implications of on- or off-budget support using this framework.

Beyond reforms to within-country resource allocation, clearer frameworks for evidence-informed prioritisation could help donors equitably and effectively prioritise investments *between* countries. A clear donor cost-effectiveness threshold would promote the concentration of funding from global health donors in the programmes and contexts in which the greatest health gains can be made with the resources available. This approach is consistent with the ethical position that all health gains should be valued equally, regardless of where occur and how they are produced.

### Options for setting thresholds

How might cost-effectiveness thresholds be set to reflect those two decision perspectives?

For national thresholds, methodological approaches to setting a threshold have been discussed extensively elsewhere. ^16^^,^^31^ In 1993, the World Bank suggested income-group-specific thresholds of US$50-200 per disability-adjusted life-year (DALY) averted. ^32^ These were superseded by the World Health Organisation’s Choosing Interventions that are Cost-Effective (WHO-CHOICE) programme, which suggested that interventions with an ICER below 3x or 1x gross domestic product (GDP) per capita could be considered “cost-effective” or “highly cost-effective”, respectively. ^23^ The use of WHO-CHOICE thresholds is now being discouraged because they appear to be too high and do not adequately reflect the resource limitations of LMICs. Indeed, WHO health economists note they were never intended to be used in the way they often were. ^14^^,^^33^ Recently, health economists have sought to clarify the theory underpinning thresholds, linking it to resource availability and local opportunity costs; with a set of estimates produced for LMICs in two papers. ^17^^,^^18^ However, only a few countries have defined an explicit cost-effectiveness threshold – for example, the UK and Thailand have, while Austria and Kenya have not. ^34^

In contrast, there is almost no literature discussing what a cost-effectiveness threshold could look like from a donor perspective. Drake (2014) outlines a case for a minimum DALY value to guide donor prioritisation. ^20^ Morton *et al.,* (2017) describe an approach towards subsidising and crowding-in services which are just cost-ineffective from a national perspective, but does not address donor-perspective thresholds. ^21^ GiveWell uses a benchmark that charity programmes should be expected to provide value at least 10 times greater than cash transfers ^35^ and Open Philanthropy requires a 1000-fold expected return on investment for their (generally higher risk and upstream) investments. ^11^

In attempting to set a threshold from a donor perspective, a first question is whether global health donors would all follow a single cost-effectiveness threshold or develop their own, agency-specific threshold. It is tempting to view each donor as having its own decision perspective and institutional mandate and therefore its own threshold. Use of numerous donor-specific thresholds could retain the advantage of improving the efficiency of each donor’s allocation between contexts, but the key challenges to coordination with national institutions and other donors would remain. To realise the benefits of improved donor harmonisation, a shared donor threshold is necessary. This threshold could be jointly agreed between donors at a country-level forum, such as a Sector Wide Approach (SWAp), which would facilitate the benefits of country-level harmonisation, but would lose the benefits of between-country resource allocation. A generalised global threshold would be required to achieve both within- and between-country benefits.

In broad terms, how could a global health donor threshold be set?

**Option 1: Notional.** Many countries and organisations use CEA to guide healthcare prioritisation without formally defining a cost-effectiveness threshold. One option is for donors to use the theoretical possibility of a separate cost-effectiveness threshold to shape policy and to clarify roles with regards to national decision-makers, without quantifying the threshold itself.

**Option 2: Supply-side.** Supply-side estimation means linking the threshold to the resources available and what they currently achieve in health production, at the margin. That is, a new investment opportunity should be more cost-effective than the next-best alternative that additional funding could support instead. If a cost-effectiveness threshold should reflect the payer’s opportunity costs, then a donor’s threshold should reflect opportunity costs at the global level, and should therefore be the same across all countries in which the donor may consider investing. It may also be possible to use statistical analysis analogous to the techniques used for estimating healthcare opportunity cost at the country level for domestic finances ^36^ to estimate the opportunity cost of marginal health aid globally.

**Option 3: Demand-side.** In contrast to the resource-linked supply-side approach, a demand-side route to setting a donor-perspective threshold could mean defining an aspirational benchmark that relevant stakeholders agree on. For example, participants in a World Health Assembly could support an aspirational declaration that all countries should be able to provide services that produce health for up to $X per DALY averted. That is, a minimum DALY value above which services should be considered worthy of investment, regardless of affordability to the national healthcare provider. Such an approach bridges the philosophical position of right-to-health advocates and technical optimisation approaches of health economists by effectively placing a minimum value on health and therefore a right to services that can produce health for this minimum standard. The drawback of the aspirational target is that it may facilitate sub-optimal allocation decisions if the demand-side aspiration is radically different from the supply-side reality. However, an important advantage of such a threshold is that it would function not only as an optimisation tool, but an advocacy goal.

## Conclusions

Economic analysis has huge potential to help donors maximise the achievement of their objectives in the face of the vast gap between need and the resources available. However, the current CEA paradigm was designed for within-country decision-making and has limited relevance to the very different environment faced by donors. In this article, we have argued that cost-effectiveness threshold(s) for global health donors should differ from thresholds for national institutions because they have different decision perspectives, budgets, and opportunity costs. We then explored some of the potential benefits of distinguishing explicitly between donor and national thresholds and briefly outlined the options for setting those thresholds. We acknowledge that the approach we propose will entail a major shift in the way donors operate by explicitly moving from maximising the direct impact and cost-effectiveness of their own investments, towards playing a supporting role to national decision-makers. There are also practical challenges in the application of this framework, including the absence of explicit national thresholds (or ‘threshold thinking’), lack of country processes and institutions to prioritise interventions and develop a core package of essential services, and the lack of cost-effectiveness evidence. Despite these challenges, developing an improved framework for priority-setting in countries where aid constitutes a substantial share of health financing could substantially strengthen health systems in those countries. For this reason, we call for further work to: i) advance methodological theory for national and donor collaboration on resource allocation, and ii) explore the political economy of such reforms.

## Data Availability

No data are associated with this article.
