# Supplementary material for: Why cost-effectiveness thresholds for global health donors should differ from thresholds for Ministries of Health (and why it matters)
Source: F1000Res. 2024 Jan 18;12:214. Originally published 2023 Feb 27. [Version 2] doi: 10.12688/f1000research.131230.2 (PMC10905028; doi:10.12688/f1000research.131230.2)
Supplement: Peer review history

# Reviewer report: Reviewer response for version 2

**DOI:** 10.5256/f1000research.161475.r238873

**Published:** 2024-02-27

**Authors:** Jessica Ochalek

**Affiliations:** 1 Centre for Health Economics, University of York, York, England, UK

I have no further comments.

Is the topic of the opinion article discussed accurately in the context of the current literature?

Yes

Are arguments sufficiently supported by evidence from the published literature?

Partly

Are all factual statements correct and adequately supported by citations?

Yes

Are the conclusions drawn balanced and justified on the basis of the presented arguments?

Partly

Reviewer Expertise:

Application of economic evaluation principles and methods to inform priority setting in healthcare, particularly in low- and middle-income countries.

I confirm that I have read this submission and believe that I have an appropriate level of expertise to confirm that it is of an acceptable scientific standard.

# Reviewer report: Reviewer response for version 1

**DOI:** 10.5256/f1000research.144045.r183976

**Published:** 2023-08-01

**Authors:** Chris Sampson

**Affiliations:** 1 Office of Health Economics, London, England, UK

I see no reason why this opinion piece should not be indexed. There are no factual errors or essential omissions. However, there are several respects in which the article may be improved, and I would encourage the authors to consider the following suggestions for a revised version. My suggestions are - roughly - in order of importance.

 1. The article proceeds on the assumption that health maximisation is the objective of (or at least an important consideration for) global health donors. It isn't clear to me that this is the case. This assumption requires i) clearer articulation as part of the authors' argumentation and ii) further discussion of its validity. 

 2. Even if health maximisation is the objective of global health donors, it isn't clear to me that the proposal summarised in Figure 1 provides a means of achieving this. It seems far more likely to me that global health donors might prefer to spend on infrastructural investments and not marginal technologies, in part because the former is more conducive to health maximisation. The authors should discuss the scenarios in which a donor's use of a threshold would and would not support health maximisation.

 3. The authors should consider (and describe) the suitability of the bookshelf analogy. In the context of low-income countries - perhaps more so than others - the bookshelf analogy is problematic. In particular, the bookshelf analogy starts to fall apart when we allow for divisibility, which is certainly a relevant consideration in countries in which providers cannot reach all patients.

 4. Some further consideration should be given (and discussion provided) to the claim that a "donor threshold should be higher". There are numerous assumptions inherent in this, and the authors have not described them. For instance, it seems to assume that resources are currently being allocated efficiently by national decision-makers. It behoves the authors to outline all of the assumptions in their claim.

 5. Throughout the article, 'thresholds' are characterised as a matter of fact, an observable quantity. This is not correct; thresholds are a policymaking tool with a primarily normative basis. For instance, the title should read that cost-effectiveness thresholds *could* or *should* differ, rather than stating that they *do* differ.

 6. The authors state that few donors rely on CEA. This raises the question of why we should care about cost-effectiveness thresholds at all! The authors should acknowledge the limited applicability and importance of their assertions in this context. Indeed, there needs to be more justification for the idea that donor cost-effectiveness thresholds should be specified at all. 

 7. The authors refer to the use of decision thresholds by Givewell and Open Philanthropy. It would be helpful to state these thresholds in the text.

 8. The claim is made that "many donors" have adopted value for money frameworks, but then only one is cited. Either more citations should be added or the claim should be altered.

Is the topic of the opinion article discussed accurately in the context of the current literature?

Yes

Are arguments sufficiently supported by evidence from the published literature?

Partly

Are all factual statements correct and adequately supported by citations?

Yes

Are the conclusions drawn balanced and justified on the basis of the presented arguments?

Yes

Reviewer Expertise:

Health economics

I confirm that I have read this submission and believe that I have an appropriate level of expertise to confirm that it is of an acceptable scientific standard.

# Response

**Published:** 2024-01-15

**Authors:** Tom Drake

**Affiliations:** Centre for Global Development, UK

We thank the reviewer for this helpful review of the article. Below we offer point-by-point responses to the feedback using [R] to denote reviewer text and [A] for author text. 

 [A] We would like to thank the reviewer for his time in providing the feedback, which has helped us to improve and clarify the manuscript. We have taken on many of the comments and adjusted the article accordingly. Point-by-point responses are offered below.

*[R] I see no reason why this opinion piece should not be indexed. There are no factual errors or essential omissions. However, there are several respects in which the article may be improved, and I would encourage the authors to consider the following suggestions for a revised version. My suggestions are - roughly - in order of importance.*

*1. The article proceeds on the assumption that health maximisation is the objective of (or at least an important consideration for) global health donors. It isn't clear to me that this is the case. This assumption requires i) clearer articulation as part of the authors' argumentation and ii) further discussion of its validity.*

 [A] Thank you for this helpful comment. To address this point, we have added a sentence that explicitly acknowledges the range of objectives that donors may pursue at the top of the section titled, “Why cost-effectiveness thresholds for global health donors should differ from thresholds for Ministries of Health”:

 The section now reads: “Donors may distribute DAH to pursue various objectives, which may include reducing preventable mortality and morbidity, protecting their own country’s health (e.g., by reducing global infectious burden of disease) or fostering wider political and economic national interests. Where health maximisation is at least one of the purposes of a donor’s DAH, and some form of cost-effectiveness thresholds are therefore potentially relevant, then we argue that the adoption of a separate threshold to national decision-makers becomes useful for the following reasons:”

*2. Even if health maximisation is the objective of global health donors, it isn't clear to me that the proposal summarised in Figure 1 provides a means of achieving this. It seems far more likely to me that global health donors might prefer to spend on infrastructural investments and not marginal technologies, in part because the former is more conducive to health maximisation. The authors should discuss the scenarios in which a donor's use of a threshold would and would not support health maximisation.*

 [A] We agree that investments like infrastructure are outside the scope of this commentary. To the extent that aid is directed towards health services (rather than health systems investments, global public goods and so on), we argue that conceptualising separate country and donor cost-effectiveness thresholds is theoretically clearer and could present opportunities for practical benefits. We have added a clarification in the second paragraph of the introduction to this effect.

 The sentence reads “While some DAH supports research, advocacy, or other catalytic activities, the vast majority supports service delivery, including both financing for specific services (which we focus on in this article) and health system strengthening such as infrastructure investments”.

*3. The authors should consider (and describe) the suitability of the bookshelf analogy. In the context of low-income countries - perhaps more so than others - the bookshelf analogy is problematic. In particular, the bookshelf analogy starts to fall apart when we allow for divisibility, which is certainly a relevant consideration in countries in which providers cannot reach all patients.*

 [A] We think that the bookshelf analogy remains relevant.  It depends on how the “books” are specified.  Additional sentence added: “Each service – or book in the bookshelf metaphor – could be defined either at the level of the national population or for specific subpopulations for whom the cost-effectiveness differs because of high need, geographical inaccessibility, or other factors.”

*4. Some further consideration should be given (and discussion provided) to the claim that a "donor threshold should be higher". There are numerous assumptions inherent in this, and the authors have not described them. For instance, it seems to assume that resources are currently being allocated efficiently by national decision-makers. It behoves the authors to outline all of the assumptions in their claim.*

 [A] We have now revised the start of the section, “Why cost-effectiveness thresholds for global health donors should differ from thresholds for Ministries of Health” to articulate more clearly the different objectives donors may pursue and our assumptions about the relevance of health maximisation (albeit not as sole criterion) to decision-making. We certainly do not intend to claim that resources are currently efficiently allocated and our arguments do not depend on such an assumption. Instead, we are arguing that increased use of thresholds could support a transition to more efficient resource allocation. We have also modified our claim in this section to argue only that the differing resource constraints mean that donor and country thresholds should be “different”; we present the wider rationale for why donor thresholds should be higher in the following section, where we are able to outline the considerations more fully.

 A revised section on why resource constraints differ now reads:

 “Different resource constraints. National decision-makers face budget constraints which reflect their country’s overall resources; the extent to which their government is able to mobilise those resources; and the prioritisation of health within national budgets. Donors, by contrast, face wholly separate resource constraints. Country donors’ budgets are constrained by their country’s resources and prioritisation of (health) aid to other countries, while private and multilateral donors’ budgets are constrained by their ability to raise revenue from government and private contributors globally. If cost-effectiveness thresholds reflect the scarcity of resources, then the  differing resource constraints imply that differing thresholds should be used.”

5. Throughout the article, 'thresholds' are characterised as a matter of fact, an observable quantity. This is not correct; thresholds are a policymaking tool with a primarily normative basis. For instance, the title should read that cost-effectiveness thresholds *could* or *should* differ, rather than stating that they *do* differ.

 [A] Thank you for raising this important point. We agree and have adjusted the language throughout, including in the title of the article.

*6. The authors state that few donors rely on CEA. This raises the question of why we should care about cost-effectiveness thresholds at all! The authors should acknowledge the limited applicability and importance of their assertions in this context. Indeed, there needs to be more justification for the idea that donor cost-effectiveness thresholds should be specified at all.*

 [A] CEA is used quite widely in several countries because the analytic community has done a good job of crafting analytic tools which are rooted in economic theory but which speak directly to the decisions faced by in-country decision makers and are contextually appropriate.  We haven’t got there for donor aid yet, we would argue because the approach has been to carry over the in-country CEA paradigm rather than figuring out how to implement those economic principles in the donor environment.  On prima facie basis, economic analysis, as the science of resource scarcity should have a greater role in the allocation of donor aid than it currently does. We hope that our commentary will encourage wider use of CEA and appropriate cost-effectiveness thresholds.

 New text: “Economic analysis has huge potential to help donors maximise the achievement of their objectives in the face of the vast gap between need and the resources available.  However, the current CEA paradigm was designed for within-country decision making and has limited read-across to the very different environment faced by donors.”

*7. The authors refer to the use of decision thresholds by Givewell and Open Philanthropy. It would be helpful to state these thresholds in the text.*

 [A] We include the following in the text: “GiveWell uses a benchmark that charity programmes should be expected to provide value at least 10 time greater than cash transfers ^29^ and Open Philanthropy require a 1000-fold expected return on investment for their (generally higher risk and upstream) investments.” These specifics come somewhat later in the text than where these donors were first mentioned, as we felt it fit better with the main flow of the text.

*8. The claim is made that "many donors" have adopted value for money frameworks, but then only one is cited. Either more citations should be added or the claim should be altered.*

 [A] Thank you for this suggestion. We have now added references to UNICEF and the Global Fund’s value-for-money frameworks, alongside the existing reference to the UK’s framework.

# Reviewer report: Reviewer response for version 1

**DOI:** 10.5256/f1000research.144045.r166801

**Published:** 2023-04-03

**Authors:** Jessica Ochalek, Karl Claxton

**Affiliations:** 1 Centre for Health Economics, University of York, York, England, UK

This opinion article aims to provide a neat and tidy solution to the problem of how donors should prioritise their funding for health both within and across countries.

 The authors start with the assumption that donors should apply a different threshold than that which should be applied by national decision makers allocating government resources for health. The latter, we agree, should reflect the marginal productivity of the healthcare system. Where there is less agreement is around whether donors should use a separate threshold at all and, if they must, what that threshold should be based upon.

 The authors rightly point out that the opportunity cost of investing in intervention A in country X is not only intervention B in country X, but also intervention C in country Y. One way to consider this is using the net health effect of the intervention within each country and across countries. (See Claxton, K. P., Ochalek, J. M., Revill, P., Rollinger, A. & Walker, D., Informing Decisions in Global Health: Cost Per DALY Thresholds and Health Opportunity Costs, Nov 2016, 4 p. Centre for Health Economics, University of York). Net health effects for each country are a function of the cost to the country (or the cost savings), health impacts and an estimate of health opportunity cost to the country of government expenditure on health.  Summing these across countries gives the global net health impact. Once this is known, the donor can select what they regard as the best (based not just the net health effects but also on their distribution across countries). 

 It may be helpful to distinguish between general budget support and off-budget support. Where funding is provided in the form of general budget support, it effectively becomes part of the government pool. Therefore, the opportunity cost of such general budget support is most closely the opportunity cost of government expenditure on health. There is no reason for a separate threshold, and donors should consider the marginal productivity of expenditure on health within the country when considering general budget support. Ceteris paribus, an expansion of the budget would result in an increase in the marginal productivity of the healthcare system.

 Where donors are providing off-budget support, given that one possibility is to give the money for the intervention to countries (on budget), donors need to consider if their off-budget project performs better or, if not, whether there are good reasons to fund it nonetheless.

 Where donors are providing off-budget support and have an exogenously fixed budget, then a supply side approach might be reasonable. This requires two things: an estimate of a “donor ICER” and an estimate of what is displaced from among existing donor-funded healthcare when the donor funds a new intervention.  Where the latter is unknown, a reasonable approach would be to rank by donor ICER. Calculating a “donor ICER” requires first calculating the global net health effects. Then calculating a “donor ICER” from the cost to the donor and the global net health.

 Where donors are providing off-budget support and have an endogenous budget, they may choose to expand their budget to continue to fund interventions until the donor’s willingness to pay for health is exhausted. Ideally, funding decisions would be taken in order of most to least cost-effective. This better aligns with the objective of health maximisation than the demand-side approach described by the authors in Option 3. Donors taking a demand side approach as described in Option 3 may fund healthcare that would be highly cost-ineffective for the country to fund. The problem with this is that donor funding can negatively impact on the efficiency of systems and sustainability of interventions through “the duplication of services, dilution and distortion of limited human and financial resources, and weak coordination between levels of care” (Barr, A., Garrett, L., Marten, R. *et al.* Health sector fragmentation: three examples from Sierra Leone. *Global Health* **15**, 8 (2019). https://doi.org/10.1186/s12992-018-0447-5). A better advocacy tool might be to illustrate the extent of possible health gains by funding healthcare closer to the margin of what is generated by the healthcare system as illustrated in Figure 1, where spending the money required to fund aid-supported services would generate more health than spending the same money to fund excluded services.

 While the authors are right that focusing national resources towards funding a core package of the most essential services would help to ensure that those services are not subject to aid volatility, a caveat to the proposal for aid supported services to be those which are just cost-ineffective for the country rather than those which are cost-effective for the country, is that many countries are not able to deliver the healthcare interventions they do provide to the full population in need of them. Adding additional services may only further detract from this ability by further diluting human resource capacity etc. Donors might instead consider spending their resources to ensure that the essential services included in the core package are accessible to everyone who requires them.

Is the topic of the opinion article discussed accurately in the context of the current literature?

Yes

Are arguments sufficiently supported by evidence from the published literature?

Partly

Are all factual statements correct and adequately supported by citations?

Yes

Are the conclusions drawn balanced and justified on the basis of the presented arguments?

Partly

Reviewer Expertise:

Application of economic evaluation principles and methods to inform priority setting in healthcare, particularly in low- and middle-income countries.

We confirm that we have read this submission and believe that we have an appropriate level of expertise to confirm that it is of an acceptable scientific standard, however we have significant reservations, as outlined above.

## References

[1] : Informing Decisions in Global /Health: Cost Per DALY Thresholds and Health Opportunity Costs [version 1; not peer reviewed] (document). *F1000Research* .2017;6(467) : 10.7490/f1000research.1113905.1 10.7490/f1000research.1113905.1 28529692

[2] : Health sector fragmentation: three examples from Sierra Leone. *Global Health* .2019;15(1) : 10.1186/s12992-018-0447-5 8 10.1186/s12992-018-0447-5 30670026 PMC6341573

# Response

**Published:** 2024-01-15

**Authors:** Tom Drake

**Affiliations:** Centre for Global Development, UK

We thank for the reviewers for their commentary in response to our article and appreciate their research in this space. Below we offer point-by-point responses to the feedback using [R] to denote reviewer text and [A] for author text. 

*[R] This opinion article aims to provide a neat and tidy solution to the problem of how donors should prioritise their funding for health both within and across countries.*

*The authors start with the assumption that donors should apply a different threshold than that which should be applied by national decision makers allocating government resources for health. The latter, we agree, should reflect the marginal productivity of the healthcare system. Where there is less agreement is around whether donors should use a separate threshold at all and, if they must, what that threshold should be based upon.*

 [A]  We build a case throughout the article (and especially in the first section titled, “Why cost-effectiveness thresholds for global health donors should differ from thresholds for Ministries of Health“) that donors should apply a different threshold from national decision-makers. We give two key reasons for the need for a different approach, namely, the differing resource constraints and the differing opportunity costs, which we explain. To make this clearer we have updated the beginning of this first section to read: “Donors may distribute DAH to pursue various objectives, which may include reducing preventable mortality and morbidity, protecting their own country’s health (e.g., by reducing global infectious burden of disease) or fostering wider political and economic national interests. Where health maximisation is at least one of the purposes of a donor’s DAH, and some form of cost-effectiveness thresholds are therefore potentially relevant, then we argue that the adoption of a separate threshold to national decision-makers becomes useful for the following reasons:”

*[R] The authors rightly point out that the opportunity cost of investing in intervention A in country X is not only intervention B in country X, but also intervention C in country Y. One way to consider this is using the net health effect of the intervention within each country and across countries. (See Claxton, K. P., Ochalek, J. M., Revill, P., Rollinger, A. & Walker, D., Informing Decisions in Global Health: Cost Per DALY Thresholds and Health Opportunity Costs, Nov 2016, 4 p. Centre for Health Economics, University of York). Net health effects for each country are a function of the cost to the country (or the cost savings), health impacts and an estimate of health opportunity cost to the country of government expenditure on health.  Summing these across countries gives the global net health impact. Once this is known, the donor can select what they regard as the best (based not just the net health effects but also on their distribution across countries).*

 [A] Thank you for this comment. We agree that net health (or monetary) benefit is one way to make cross-country comparisons. However, a key limitation to this approach is that the conversion factor between money and health in net health calculations, the health production function, is typically the cost-effectiveness threshold. If the CET should reflect the total resource envelope, then it will also be a product of the donor allocation decision as well as - in this use case - a determinant of it.

 Secondly, a key element of our proposed approach in the section "Why does this matter" is the process of negotiation and partnership that can result from explicit negotiation of who will fund which services and why. A joint threshold, concealed in a net health benefit calculation does not support such an approach.

*[R] It may be helpful to distinguish between general budget support and off-budget support. Where funding is provided in the form of general budget support, it effectively becomes part of the government pool. Therefore, the opportunity cost of such general budget support is most closely the opportunity cost of government expenditure on health. There is no reason for a separate threshold, and donors should consider the marginal productivity of expenditure on health within the country when considering general budget support. Ceteris paribus, an expansion of the budget would result in an increase in the marginal productivity of the healthcare system.*

**

*Where donors are providing off-budget support, given that one possibility is to give the money for the intervention to countries (on budget), donors need to consider if their off-budget project performs better or, if not, whether there are good reasons to fund it nonetheless.*

**

*Where donors are providing off-budget support and have an exogenously fixed budget, then a supply side approach might be reasonable. This requires two things: an estimate of a “donor ICER” and an estimate of what is displaced from among existing donor-funded healthcare when the donor funds a new intervention.  Where the latter is unknown, a reasonable approach would be to rank by donor ICER. Calculating a “donor ICER” requires first calculating the global net health effects. Then calculating a “donor ICER” from the cost to the donor and the global net health.*

 [A] Thank you for raising the important practical consideration of how the proposed framework might be affected by the practical approach to financing and delivery. We agree that these are important considerations and in fact are the subject of ongoing follow up work. We disagree that our proposed approach is incompatible with on-budget support. First, tracking and monitoring of on-budget support is technically feasible (if burdensome), enabling donor funding to be earmarked for specific services while being channelled through public systems. Second, thresholds could be used during budget formulation to negotiate and agree levels of on budget support to provide, even if contributions are not then traced through public financial systems. Thirdly, when a donor provides on-budget support to a country, the opportunity cost of that support is the value that could have been gained had that DAH been invested in either a project in that country OR in a project or on-budget support in another country. So the fact that for donors the opportunity cost of on-budget support is not restricted to the recipient country implies a different decision threshold.

 Public financial management considerations will be the subject of future publications and are beyond the scope of this commentary. We have added a note to the article that “Further work is needed to consider the public financial management implications of on- or off-budget support using this framework.”

*[R] Where donors are providing off-budget support and have an endogenous budget, they may choose to expand their budget to continue to fund interventions until the donor’s willingness to pay for health is exhausted. Ideally, funding decisions would be taken in order of most to least cost-effective. This better aligns with the objective of health maximisation than the demand-side approach described by the authors in Option 3. Donors taking a demand side approach as described in Option 3 may fund healthcare that would be highly cost-ineffective for the country to fund. The problem with this is that donor funding can negatively impact on the efficiency of systems and sustainability of interventions through “the duplication of services, dilution and distortion of limited human and financial resources, and weak coordination between levels of care” (Barr, A., Garrett, L., Marten, R. et al. Health sector fragmentation: three examples from Sierra Leone. Global Health **15**, 8 (2019).* *https://doi.org/10.1186/s12992-018-0447-5* *). A better advocacy tool might be to illustrate the extent of possible health gains by funding healthcare closer to the margin of what is generated by the healthcare system as illustrated in Figure 1, where spending the money required to fund aid-supported services would generate more health than spending the same money to fund excluded services.*

 [A] Thank you for this comment. We do not promote Option 3 over and above Options 1 and 2, but rather, attempt to describe the strengths and weaknesses of each. We agree with the potential risks of a demand-derived threshold and acknowledge them in the article, where we write: "The drawback of the aspirational target is that it may allow sub-optimal allocation decisions if the demand-side aspiration is radically different from the supply-side reality." We also allude to the potential for endogenous budgets and the potential for aid budgets to be expanded in line with aspirational targets, that is, indications of what donors’ collective willingness-to-pay should be. At the beginning of the section on options for setting thresholds, we recognise the historic and ongoing issues with using the 1x and 3x GDP thresholds for optimising resource allocation from a country decision-maker perspective. For completeness in describing the options available for setting a donor perspective threshold, it makes sense to include both supply and demand options and the significant differences between the country and donor perspective thresholds, both in terms of the cohesiveness of the budget/payer and how the threshold could inform resource allocation and mobilisation.

   

*[R] While the authors are right that focusing national resources towards funding a core package of the most essential services would help to ensure that those services are not subject to aid volatility, a caveat to the proposal for aid supported services to be those which are just cost-ineffective for the country rather than those which are cost-effective for the country, is that many countries are not able to deliver the healthcare interventions they do provide to the full population in need of them. Adding additional services may only further detract from this ability by further diluting human resource capacity etc. Donors might instead consider spending their resources to ensure that the essential services included in the core package are accessible to everyone who requires them.*

 [A] We agree that ensuring effective quality delivery of the core package is a priority and that development partners may have an important role to play in achieving this goal, including by financing (or even directly delivering) these services for a period. However, as part of the development process, countries must be supported to take on the responsibility for financing and delivering the highest priority services and this could easily happen while total budget envelope is still low enough to require financial support to achieve an acceptable essential package of services. In short, we agree that the approach should be a new deal or compact to strengthen country processes for prioritisation, financing and delivery, place countries in the driving seat of the prioritisation process and (implicitly or explicitly) link additional financing for services to those at the margin as defined by countries, not to funder priorities.
